# Economic burden of multiple sclerosis in a population with low physical disability

**DOI:** 10.1186/s12889-019-6907-x

**Published:** 2019-05-20

**Authors:** José M. García-Domínguez, Jorge Maurino, María L. Martínez-Ginés, Olga Carmona, Ana B. Caminero, Nicolás Medrano, Elena Ruíz-Beato, for the W-IMPACT Clinical Investigators, Adrián Ares, Carmen Arnal, Ana B. Caminero, María Carcelén, Olga Carmona, José M. García-Domínguez, María L. Martínez-Ginés, Pablo Eguía, María del Carmen Fernández, Ricardo Ginestal, Laura Lacruz, Miguel Llaneza, Carlos López de Silanes, Gisela Martín, Laura Navarro, Beatriz Romero, María Seral, Myriam Solar

**Affiliations:** 1 Department of Neurology, Hospital Universitario Gregorio Marañón, Madrid, Spain; 2 Medical Department, Roche Farma, Ribera del Loira, 50, 28042 Madrid, Spain; 3 Department of Neurology, Hospital de Figueres, Figueres, Spain; 4 Department of Neurology, Hospital Nuestra Señora de Sonsoles, Complejo Asistencial de Ávila, Ávila, Spain; 5 Health Economics and Outcomes Research Unit, Roche Farma, Madrid, Spain

**Keywords:** Multiple sclerosis, Disability, Costs, Burden of illness, Caregiver

## Abstract

**Background:**

In multiple sclerosis (MS), half of affected people are unemployed within 10 years of diagnosis. The aim of this study was to assess the economic impact of MS in adult subjects with relapsing-remitting MS (RRMS) and primary progressive MS (PPMS).

**Methods:**

A multicenter, non-interventional, cross-sectional study was conducted. The Expanded Disability Status Scale (EDSS) and the 23-item Multiple Sclerosis Work Difficulties Questionnaire (MSWDQ-23) were used to assess disability and work performance, respectively. Only indirect costs were considered using the human capital method, including work costs. Professional support costs and informal caregivers’ costs were also estimated.

**Results:**

A total of 199 subjects were studied (mean age: 43.9 ± 10.5 years, 60.8% female, 86.4% with RRMS). Median EDSS score was 2.0 (interquartile range: 1.0–3.5) and median MSWDQ-23 total score was 31.5 (15.2, 50.0). The number of employed subjects decreased after MS diagnosis from 70.6 to 47.2%, and the number of retired people increased (23.6%). Mean age of retirement was 43.6 ± 10.5 years. Ten percent of the population had sick leaves (absenteeism was seen in 90.9% of the student population and 30.9% of the employed population). Professional support in their daily life activities was needed in 28.1% of subjects. Costs for sick leave, work absenteeism, premature retirement and premature work disability/pensioner were €416.6 ± 2030.2, €763.4 ± 3161.8, €5810.1 ± 13,159.0 and €1816.8 ± 9630.7, respectively. Costs for professional support and informal caregiving activities were €1026.93 ± 4622.0 and €1328.72, respectively.

**Conclusions:**

MS is responsible for a substantial economic burden due to indirect and informal care costs, even in a population with low physical disability.

## Background

Multiple sclerosis (MS) is a chronic autoimmune neurological disease with a high impact on the health-related quality of life of individuals, their families and society [1–5]. MS is typically diagnosed in young, active people between 20 and 40 years of age [6]. Therefore, the disease may hinder ability to maintain studies and work [1–4]. According to the Global MS Employment Report 2016, 43% of unemployed people with MS quit their employment in the first 3 years after diagnosis and 62% stated that fatigue was the main reason [7]. In addition, pwMS will require caregiving due to disability progression, mostly provided by informal caregivers, such as partners or other relatives [5].

Total average annual costs were €41,212 ± €18,761 in an observational study of 1152 pwMS and 265 caregivers conducted in 19 countries [3]. Over half of total costs were associated with direct medical costs, followed by indirect costs (€17,492) and direct non-medical costs (€2157). In some European countries, MS caregivers provide 150 h a month of care to pwMS, the equivalent of full-time employment [1]. The mean annual cost per patient in Spain was €30,050 [8]. Sicras et al. found that only 30% of MS people were employed or self-employed [9]. More than 30% of pwMS had an early retirement, in most cases due to disability. Indirect costs in Spain accounted for between 24 and 35% of disease costs, increasing as disability advanced, and reaching over €15,779 in subjects essentially restricted to a wheelchair [8]. However, available data related to the economic burden of MS, especially limitations in work activities and job performance remains limited. The objective of this study was to assess the indirect costs and informal care costs of MS in a sample of pwMS in Spain.

## Methods

### Study design

A non-interventional, cross-sectional study in subjects with relapsing-remitting (RRMS) and primary-progressive MS (PPMS) according to McDonald 2010 criteria was conducted (W-IMPACT study) [10, 11]. Nineteen MS units across Spain were invited to participate for their good clinical practice and expertise conducting non-interventional research by the study steering committee. Investigators included the first ten consecutive subjects that met the inclusion criteria. Competitive recruitment was established among centers.

### Clinical variables

Disability was assessed using the Expanded Disability Status Scale (EDSS) [12]. Total score ranges from 1 (no disability) to 10 (death due to MS). The 29-item Multiple Sclerosis Impact Scale (MSIS-29) was used to measure the physical and the psychological impact of MS from the patients’ perspective [13]. Physical and psychological impact scores range from 20 to 80 and from 9 to 36, respectively. Higher scores indicate greater impact. Work performance related to physical, cognitive and psychological dimensions was studied with the 23-item Multiple Sclerosis Work Difficulties Questionnaire (MSWDQ-23) [11, 14]. Scores range from 0 to 100 with higher values indicating greater workplace problems [14]. When possible, caregivers completed the Caregiver Strain Index (CSI), a self-rated questionnaire for measuring the perceived level of burden [15]. A total score of seven or higher indicates a high level of caregiver burden.

### Healthcare resources and costs

Cost descriptions were summarized in the student and employed populations, separately. Only indirect and informal care costs of MS were included in the study analysis. Indirect costs were calculated using the method of human capital where the production of a person is valued at the market price. Indirect costs for employed people were considered as the gross salary per hour, and for unemployed subjects as the minimum salary per hour, in order to avoid underestimating the indirect costs. The cost of pwMS who had a sick leave during the last year was calculated by multiplying the number of days lost by the salary indicated in the employment status section. The cost of lost study hours in people who were studying at the time of the study was calculated as the lost hours per week multiplied by 52. Costs of premature retirement and premature work disability/pensioner were calculated as the indicated annual salary.

PwMS who needed to be accompanied by a formal caregiver were considered in the study by also taking into account the type of resource used, the number of hours per week and the number of weeks they required the resource in the last year. Cost was calculated for each service using the indicated cost multiplied by 12. Costs of informal caregivers were calculated by multiplying the number of hours of work spent without working or with reduced productivity and the days of absence from work by the salary.

Additional costs such as direct non-healthcare costs (transportation and home or car modifications) and direct healthcare costs (use of non-reimbursed devices and aids, private physiotherapy and private psychological treatment) were calculated by multiplying the resources used per subject by their unit cost. All these unit costs were obtained from the health costs database eSalud [16]. All costs were calculated in euros per subject per year based on EDSS score and updated with the Consumer Price Index (CPI).

### Statistical analysis

Continuous variables were described by the number of available values, mean, standard deviation, and median, 25th percentile (P25), 75th percentile (P75), minimum and maximum values. Categorical variables were described as the total number of available values and relative percentage per subgroup of interest.

## Results

A total of 199 pwMS were included in the study. Subjects were predominantly female (60.2%), with a diagnosis of RRMS (86.1%) and a mean age of 43.9 ± 10.5 years. The median EDSS score was 2.0 (interquartile range 1.0, 3.5). Table 1 shows the main demographic and clinical characteristics of the sample.

**Table 1:** Demographic and clinical characteristics

	Total (*n* = 199)
Age (years); mean (SD)	43.9 (10.5)
Gender (female); n (%)	121 (60.8%)
Educational level; n (%)	
-Primary	32 (16.1%)
-Secondary	88 (44.2%)
-University/Postgraduate studies	79 (39.7%)
Type of MS; n (%)	
-RRMS	172 (86.4%)
-PPMS	27 (13.6%)
Time since diagnosis (years); mean (SD)	9.6 (7.2)
Number of relapses in the last 2 years; mean (SD)	0.6 (0.9)
Time since last relapse (months); mean (SD)	53.9 (56.1)
Patients on DMT; n (%)	160 (80.4%)
EDSS score; median (IRQ)	2.0 (1.0, 3.5)
MSWDQ-23 total score; median (IRQ)	31.5 (15.2, 50.0)

*DMT* Disease-modifying therapies, *EDSS* Expanded Disability Status Scale, *IRQ* Interquartile range, *MS* Multiple sclerosis, *MSWDQ-23* 23-item Multiple Sclerosis Working Difficulties, *PPMS* Primary progressive multiple sclerosis, *RRMS* Relapsing-remitting multiple sclerosis, *SD* Standard deviation

**Fig. 1:**
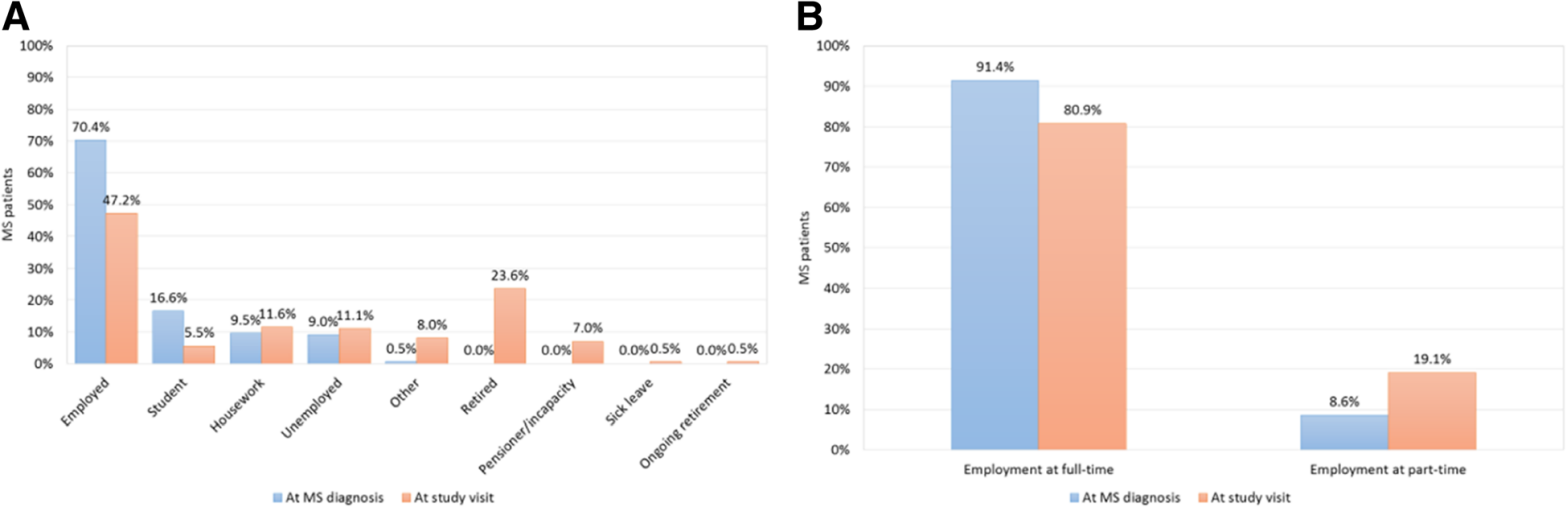
Employment status: At MS diagnosis (**a**) and At study visit (**b**)

**Fig. 2:**
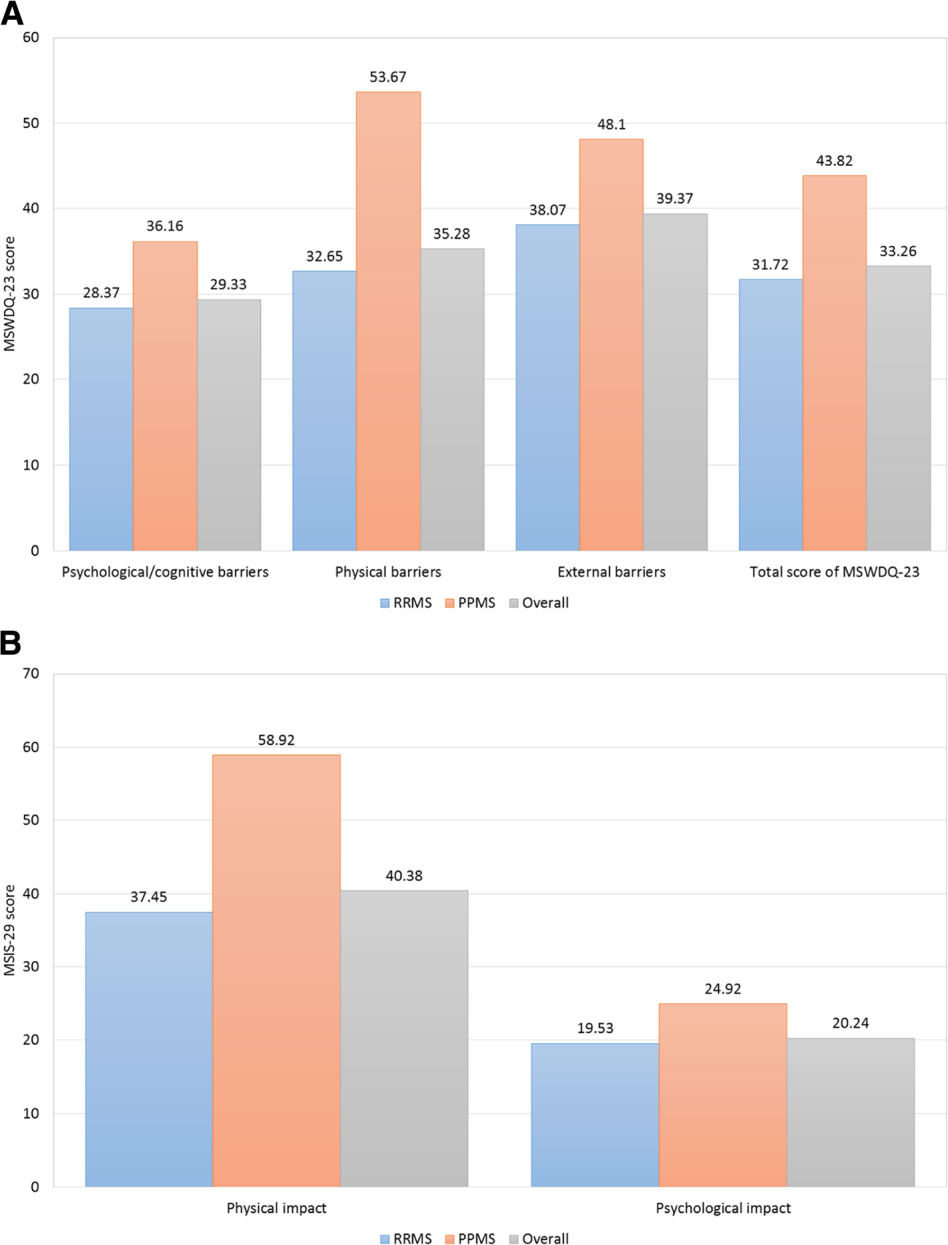
Impact of MS on patients: MSWDQ-23 (**a**) and MSIS-29 (**b**) questionnaires

Most subjects (70.4%) were employed at MS diagnosis, but only 47.2% were employed at the time of the study visit (Fig. 1). Mean time between MS diagnosis and the study visit was 9.6 years (SD 7.2). The mean age for retirement was 43.6 ± 10.5 years, with disease progression as the main reason (95.7%). According to the results collected from the MSWDQ-23 and MSIS-29 questionnaires, the main impact of MS on subjects’ activities was due to external and physical barriers, especially in those patients with PPMS (Fig. 2).

During the last year prior to the study visit, the mean free time that subjects had was 45.7 ± 35.8 h/week. PwMS dedicated 35.5 ± 17.9 h/week to their studies and 36.2 ± 10.8 h/week to their work activity. Sick leaves were reported in 10.1% of the total MS population and absenteeism was seen in 90.9% of the student population and 30.9% of the employed population (Table 2).

**Table 2:** Impact of MS on academic and work activity during the last year

	Student pwMS				
	EDSS 0–1 (*n* = 7)	EDSS 1.5–3.0 (*n* = 4)	EDSS 3.5–5.5 (*n* = 0)	EDSS ≥ 6 (*n* = 0)	Overall (*n* = 11)
Academic absenteeism; n (%)	0 (0%)	1 (25.0%)	0 (0.0%)	0 (0%)	1 (9.1%)
Time off study (hours/year); mean (SD)	0 (0)	13.0 (26.0)	0 (0)	0 (0)	4.7 (15.7)
Lost time percentage in a week compared to a normal week (hours); mean (SD)	0 (0)	0.4 (0.8)	0 (0)	0 (0)	0.2 (0.5)
	Employed pwMS				
	EDSS 0–1 (*n* = 45)	EDSS 1.5–3.0 (*n* = 39)	EDSS 3.5–5.5 (*n* = 10)	EDSS ≥ 6 (*n* = 0)	Overall (*n* = 94)
Sick leave; n (%)	5 (11.1%)	11 (28.2%)	4 (40.0%)	0 (0%)	20 (21.3%)
Time on sick leave (days); mean (SD)	1.9 (6.4)	28.9 (74.0)	37.7 (93.3)	0 (0)	16.9 (57.6)
Work absenteeism; n (%)	12 (26.7%)	14 (35.9%)	3 (30.0%)	0 (0%)	29 (30.9%)
Time lost from work (hours/year); mean (SD)	101.7 (344.9)	281.9 (618.3)	312.0 (914.9)	0 (0)	198.8 (549.8)
Lost time percentage in a week compared to a normal week (hours); mean (SD)	5.2 (16.2)	14.6 (30.5)	11.0 (31.4)	0 (0)	9.7 (24.9)
	Premature retirement and work disability				
	EDSS 0–1 (*n* = 67)	EDSS 1.5–3.0 (*n* = 77)	EDSS 3.5–5.5 (*n* = 34)	EDSS ≥ 6 (*n* = 21)	Overall (*n* = 199)
Premature retirement due to MS; n (%)	4 (6.0%)	15 (19.5%)	10 (29.4%)	16 (76.2%)	45 (22.6%)
Premature work disability/pensioner due to MS; n (%)	0 (0%)	7 (9.1%)	5 (14.7%)	2 (9.5%)	14 (7.0%)

*EDSS* Expanded Disability Status Scale, *MS* Multiple sclerosis, *pwMS* People with multiple sclerosis, *SD* Standard deviation

About a third (36.2%) of pwMS went to the study visit accompanied by a formal caregiver and almost half (44.7%) by an informal one (Table 3). The most frequent informal caregiving was provided by a direct relative (55.82%) (Table 3). The mean time spent on caregiving activities for these informal caregivers was: husband/wife (212.14 ± 516.86 h/year); other relative (154.19 ± 503.18 h/year), and neighbor (3.02 ± 21.97 h/year). A total of 72 caregivers completed the CSI (mean total score was 3.9 ± 3.4). The prevalence of a high level of strain was 23.6% (*n* = 17).

**Table 3:** Professional and informal support

	Professional support				
	EDSS 0–1 (*n* = 67)	EDSS 1.5–3.0 (*n* = 77)	EDSS 3.5–5.5 (*n* = 34)	EDSS ≥ 6 (*n* = 21)	Overall (*n* = 199)
Formal caregiver at study visit; n (%)	15 (22.4%)	25 (32.5%)	20 (58.8%)	12 (57.1%)	72 (36.2%)
Support staff (professionals or transportation) paid by the patient; n (%)	5 (7.5%)	25 (32.5%)	14 (41.2%)	12 (57.1%)	56 (28.1%)
Type of professional; n (%)					
- Caregiver	0 (0%)	0 (0%)	1 (2.9%)	0 (0%)	1 (0.5%)
- Physiotherapist	2 (3.0%)	15 (19.5%)	10 (29.4%)	5 (23.8%)	32 (16.1%)
- Person to do housework	2 (3.0%)	8 (10.4%)	3 (8.8%)	5 (23.8%)	18 (9.0%)
- Private ophthalmologist	0 (0%)	2 (2.6%)	1 (2.9%)	1 (4.8%)	4 (2.0%)
- Private neurologist	0 (0%)	1 (1.3%)	0 (0%)	0 (0%)	1 (0.5%)
- Private psychologist	2 (3.0%)	5 (6.5%)	1 (2.9%)	0 (0%)	8 (4.0%)
- Taxi driver	1 (1.5%)	4 (5.2%)	6 (17.6%)	4 (19.0%)	15 (7.5%)
- Other	0 (0%)	7 (9.1%)	5 (14.7%)	1 (4.8%)	13 (6.5%)
	Informal support				
	EDSS 0–1 (*n* = 67)	EDSS 1.5–3.0 (*n* = 77)	EDSS 3.5–5.5 (*n* = 34)	EDSS ≥ 6 (*n* = 21)	Overall (*n* = 199)
Informal caregiver at study visit; n (%)	13 (19.4%)	30 (39.0%)	28 (82.4%)	18 (85.7%)	89 (44.7%)
Relationship to patient; n (%)					
- Husband/wife	8 (11.9%)	19 (24.7%)	21 (61.8%)	12 (57.1%)	60 (30.2%)
- Other relative	5 (7.5%)	18 (23.4%)	19 (55.9%)	9 (42.9%)	51 (25.6%)
- Neighbor	1 (1.5%)	2 (2.6%)	1 (2.9%)	1 (4.8%)	5 (2.5%)
- Volunteer	0 (0%)	0 (0%)	0 (0%)	1 (4.8%)	1 (0.5%)
- Other	1 (1.5%)	1 (1.3%)	1 (2.9%)	0	3 (1.5%)
	Use of non-reimbursed devices				
	EDSS 0–1 (*n* = 67)	EDSS 1.5–3.0 (*n* = 77)	EDSS 3.5–5.5 (*n* = 34)	EDSS ≥ 6 (*n* = 21)	Overall (*n* = 199)
Use of non-reimbursed devices; n (%)	1 (1.5%)	11 (14.3%)	15 (44.1%)	16 (76.2%)	43 (21.6%)
Type of device; n (%)					
- Wheelchair	0 (0%)	0 (0%)	1 (2.9%)	3 (14.3%)	4 (2.0%)
- Crutch/walking stick	0 (0%)	3 (3.9%)	7 (20.6%)	11 (52.4%)	21 (10.6%)
- Toilet seats or grab bars	0 (0%)	2 (2.6%)	4 (11.8%)	5 (23.8%)	11 (5.5%)
- Home adaptation	0 (0%)	6 (7.8%)	5 (14.7%)	5 (23.8%)	16 (8.0%)
- Vehicle adaptation	0 (0%)	1 (1.3%)	2 (5.9%)	3 (14.3%)	6 (3.0%)
- Articulated bed	0 (0%)	0 (0%)	0 (0%)	2 (9.5%)	2 (1.0%)
- Other	1 (1.5%)	1 (1.3%)	5 (14.7%)	6 (28.6%)	13 (6.5%)

*EDSS* Expanded Disability Status Scale, *MS* Multiple sclerosis

Over a quarter (28.1%) of pwMS needed paid professional support to maintain their daily life activities, mainly a person to do housework (0.5 ± 2.3 h/week and 4.4 ± 14.5 weeks/year) and a physiotherapist (0.4 ± 1.0 h/week and 5.3 ± 14.8 weeks/year) (Table 4).

**Table 4:** Description of number of hours per week and number of weeks that patient needed help from third parties (non-relatives), professionals or transportation paid by patient for a reason related to MS in the last year

	Hours per week Mean (SD)	Weeks per year Mean (SD)
Type		
Caregivers	0.43 (6.03)	0.26 (3.69)
Physiotherapist	0.36 (0.97)	5.25 (14.84)
Person to do housework	0.51 (2.33)	4.44 (14.50)
Private ophthalmologist	0.02 (0.17)	0.27 (3.69)
Private neurologist	0.01 (0.07)	0.26 (3.69)
Private psychologist	0.09 (0.63)	1.43 (8.15)
Other	0.21 (1.35)	2.47 (10.63)
Valid n	199	199
Missing n	0	0

The mean (SD) annual cost associated with sick leaves due to MS was €416.6 ± 2030.2, with work absenteeism due to MS accounting for €763.4 ± 3161.8, premature retirement due to MS for €5810.1 ± 13,159, and premature work disability/ pensioner for €1816.8 ± 9630.7 per patient (Table 5). The sum of all these indirect costs resulted in an average cost per patient per year of €8806.9. Considering caregiving activities, professional staff cost was €1026.9 ± 4622.0 and informal caregiving activities had a cost of €301.7 ± 1160.0, resulting in a total average cost for caregiving activities of €1328.7. Finally, the mean cost of MS devices needed by pwMS was €736.6 ± 2756.4. All these costs were considered to be average for MS subjects.

**Table 5:** MS costs by EDSS score

	EDSS 0–1 (*n* = 67)	EDSS 1.5–3.0 (*n* = 77)	EDSS 3.5–5.5 (*n* = 34)	EDSS ≥6 (*n* = 21)	Overall (*n* = 199)
Sick leave; mean (SD)	80.25 (334.38)	729.52 (2707.92)	627.91 (2646.44)	0 (0)	416.58 (2030.18)
Annual cost (work absenteeism); mean (SD)	510.89 (2089.42)	1280.35 (4279.86)	561.76 (2810.88)	0 (0)	763.40 (3161.83)
Annual cost of premature retirement due to MS; mean (SD)	1006.27 (4144.32)	5302.33 (12,546.38)	8169.30 (16,952.79)	19,178.96 (17,600.87)	5810.12 (13,158.95)
Annual cost of premature work disability/pensioner due to MS; mean (SD)	0 (0)	2941.80 (14,243.37)	2926.08 (7568.11)	1692.58 (5896.10)	1816.83 (9630.69)
Professional staff cost; mean (SD)	290.15 (1576.30)	679.64 (1406.23)	3246.71 (10,532.42)	1057.14 (1442.27)	1026.93 (4622.00)
Informal support cost; mean (SD)	125.76 (771.81)	320.97 (1274.07)	575.22 (1329.16)	350.36 (1427.77)	301.79 (1160.04)
Use of non-reimbursed devices; mean (SD)	0.39 (3.18)	508.95 (2409.37)	1195.59 (3543.34)	3177.05 (4880.50)	736.60 (2756.47)

*EDSS* Expanded Disability Status Scale, *MS* Multiple sclerosis, *SD* Standard deviation

## Discussion

The economic cost of MS is largely driven by indirect costs that are linked to early and very high unemployment rates [1–4, 17, 18]. As stated earlier, this study provides a better understanding of the impact of MS on Spanish patients’ working activity, allowing for an updated estimation of the indirect costs of MS. Our study shows an employment rate of 47.2% and a mean age of retirement of 43.6 years in a clinically stable sample of 199 pwMS mostly with a low level of physical disability (median EDSS score of 2.0). Almost a quarter (22.4%) of pwMS retired early because of the condition and 7% received an incapacity benefit or pension due to MS. Ten percent of the population had sick leaves (absenteeism was seen in 90.9% of the student population and 30.9% of the employed population). Costs associated with work limitations to employed subjects were almost seven times greater than costs from professional support and 30 times greater than costs from informal caregiving. Among all costs, those with the higher value are the costs associated with premature retirement according to our sample population (women fully incorporated into working life). These results are consistent with several prior studies. Kobelt et al. found that productivity losses and informal care dramatically increased from €593 at early-stage disease to nearly €34,228 at EDSS scores > 7 [19]. Subsequently, the TRIBUNE study also showed the correlation between disability and economic impact [20]. The overall annual indirect costs associated with MS were estimated at between €207 and €440 million due to productivity losses caused by lost work hours and early retirement (27 and 33% of the total cost, respectively) [21, 22].

A high rate of unemployment also occurs at a level of disability which is typically not associated with overt physical disability [1, 17]. Less visible symptoms and difficulties including cognitive impairment, fatigue, anxiety and depression are reasons for low productivity and unemployment at low EDSS scores. Kobelt et al. also found an employment rate of 45% in a sample of 462 pwMS in Spain with an EDSS score between 0 and 3 [23]. Fatigue and cognitive complaints were found in 92 and 64% of participants, respectively. Fatigue and cognitive disturbances are much more common than mobility problems in people who are working [8, 19]. MS-related productivity loss due to presenteeism was three times that of absenteeism in a study with 740 MS employees conducted in Australia [4]. The mean total work productivity loss was 2.5 days (14.2% loss in productive time), based on an absenteeism of 0.6 days (3.4%) and a presenteeism of 1.9 days (10.8%), leading to a €4578 loss per person annually. Work productivity was determined mostly by fatigue, cognitive impairment, pain and sensory symptoms [4].

There is increasing evidence to advocate the use of more effective therapies at earlier stages of the disease [24–27]. The IMPrESS study showed that pwMS treated earlier in the course of the disease showed a trend towards lower total (€39,037 vs €42,996), indirect (€15,733 vs €18,934) and disease-modifying therapy (€19,364 vs €20,491) costs and a better health-related quality of life status (0.62 vs 0.56; *p* < 0.01) compared to those receiving late treatment [3]. Chen et al. found that pwMS receiving high-efficacy disease-modifying therapy reported significant increases in amount of work, work attendance and work productivity compared with those using first-generation injectable treatments [27].

Our study has several limitations. Recall bias is a common concern of studies using self-reported data. The relatively small sample size and the cross-sectional design could be additional limiting factors of this study. A salary was set based on the range indicated by the participant (mid-point). For cases where only the salary at the time of diagnosis was available, the point of the range was adjusted using the CPI for the respective year. Another limitation is not having considered the time lost due to disease for pwMS without paid work, since it was unknown during the study.

## Conclusions

MS has classically been reported as being responsible for high indirect costs and other substantial economic burden in Spain. Symptom severity has strong impact on both work productivity and workforce participation. This study shows that indirect and caregiving costs are incurred even at low levels of physical disability. Effective therapeutic interventions to improve the management of early symptoms as well as implementing workplace strategies focused on job retention may be essential to decrease the high economic burden of MS.

## Abbreviations

CSI: Caregiver Strain Index
EDSS: Expanded Disability Status Scale
IQR: Interquartile range
MS: Multiple sclerosis
MSIS-29: 29-item Multiple Sclerosis Impact Scale
MSWDQ-23: 23-item Multiple Sclerosis Work Difficulties Questionnaire
PPMS: Primary progressive multiple sclerosis
pwMS: People with multiple sclerosis
RRMS: Relapsing-remitting multiple sclerosis
SD: Standard deviation
