# Correction to: Economic burden of multiple sclerosis in a population with low physical disability

**DOI:** 10.1186/s12889-019-7275-2

**Published:** 2019-07-08

**Authors:** José M. García-Domínguez, Jorge Maurino, María L. Martínez-Ginés, Olga Carmona, Ana B. Caminero, Nicolás Medrano, Elena Ruíz-Beato, for the W-IMPACT Clinical Investigators

**Affiliations:** 1 Department of Neurology, Hospital Universitario Gregorio Marañón, Madrid, Spain; 2 Medical Department, Roche Farma, Ribera del Loira, 50, 28042 Madrid, Spain; 3 Department of Neurology, Hospital de Figueres, Figueres, Spain; 4 Department of Neurology, Hospital Nuestra Señora de Sonsoles, Complejo Asistencial de Ávila, Ávila, Spain; 5 Health Economics and Outcomes Research Unit, Roche Farma, Madrid, Spain

**Correction to: BMC Public Health**

**https://doi.org/10.1186/s12889-019-6907-x**

It has been highlighted that the original article [1] contained a mistake in the ‘Results’ section, specifically in the percentages of female subjects and those with diagnosis of RRMS. Please note that this mistake has only been present in the ‘Results’ section, the Abstract and Table 1 remain unchanged. This article shows the incorrect and correct version of the percentages.

**Incorrect**:

Subjects were predominantly female (60.2%), with a diagnosis of RRMS (86.1%) and a mean age of 43.9 ± 10.5 years.

**Correct:**

Subjects were predominantly female (60.8%), with a diagnosis of RRMS (86.4%) and a mean age of 43.9 ± 10.5 years.
